# Patterns of foot-and-mouth disease virus detection in environmental samples in an endemic setting

**DOI:** 10.3389/fvets.2023.1157538

**Published:** 2023-06-16

**Authors:** Sarah R. Mielke, Sevidzem Lendzele, Amy H. Delgado, Mamoudou Abdoulmoumini, Simon Dickmu, Rebecca Garabed

**Affiliations:** ^1^Department of Veterinary Preventive Medicine, The Ohio State University College of Veterinary Medicine, Columbus, OH, United States; ^2^United States Department of Agriculture’s Animal and Plant Health Inspection Service (APHIS), Fort Collins, CO, United States; ^3^Transmissible Diseases Ecology Laboratory, Department of Environmental Health, Faculty of Technology and Health Management, Université Libreville Nord, Libreville, Gabon; ^4^School of Veterinary Science and Medicine, University of Ngaoundéré, Ngaoundéré, Adamawa, Cameroon; ^5^The National Veterinary Laboratory (LANAVET), Garoua North, Cameroon; ^6^University of Bamenda, Bambili, Cameroon

**Keywords:** viral RNA detection, endemic FMD, environment, disease modeling, Bayesian analysis

## Abstract

Foot-and-Mouth Disease virus (FMDV) is endemic in several regions and is a virus that can persist in the environment dependent on pH, relative humidity (RH), temperature, and matrix (i.e., soil, water, or air). Our previously published analysis of available viral persistence data showed that persistence is likely affected by interactions between RH, temperature, and matrix. Understanding these relationships will aid efforts to eliminate FMD, which has significant impacts on economies and food security. In Cameroon, West Africa, the livestock system consists of mobile (transhumant), transboundary trade and sedentary herds. Studying this system can provide information about the patterns of environmental detection of FMDV RNA that may influence approaches to virus elimination on premises during an outbreak. To improve our understanding of these patterns, we collected samples from individuals, vehicles, and along cattle pathways at three sedentary herds beginning on day one of owner-reported outbreaks, ending by day 30, and tested for the presence of FMD viral RNA using rRT-PCR. Our analysis suggests that detection decreases in soil surface samples with increased distance from herd and time from the first report of disease. Whereas time but not distance decreases detection in air samples. Interaction of RH and temperature suggests increased detection at high temperatures (>24°C) and RH (>75%), providing us with new information about the patterns of FMD viral RNA detection in and around cattle herds that could help to inform targeted virus elimination strategies, such as location and application of disinfectants.

## 1. Introduction

Environmental detection of viral components using rRT – PCR is a way for outbreak investigators, researchers, and planners to evaluate patterns of detection in and around disease outbreak premises. How best to use this information is the question? This method detects viral components but does not tell us whether the virus is infectious without using methods such as virus isolation. Understanding where foot-and-mouth disease viral (FMDV) RNA may be detected in endemic settings, in the absence of vaccination or cleaning and disinfection, provides baseline information to support surveillance and virus elimination strategies that are tailored to the virus and the environment. While approaches to cleaning and disinfecting in developing countries may be extrapolated from industry best practices, data to support environmental sampling approaches are sparse (6, 10, 23). In particular, the interplay between routine environmental degradation of FMDV and the ability to detect viral RNA in extensive production settings has not been evaluated. Therefore, the purpose of this study was to evaluate the detection of FMDV by PCR testing in the environment around infected cattle herds under natural conditions. This information will help support the development of environmental sampling approaches and guide further investigation on this topic relevant to mitigation strategies.

FMDV can be found in all secretions and excretions of infected animals, and the virus has been shown to persist in the environment, dependent on a range of pH, relative humidity (RH), and temperature (1–3, 5, 14, 19, 20, 25, 29, 33, 39). Furthermore, interactions between RH, temperature, and the type of matrix FMD viral RNA is shed upon, such as soil, water, air, or fomites is likely to affect where FMD viral RNA presence is detected in the environment (23). Environmental transmission of FMDV has been shown to occur, and a recent experimental study by Colenutt et al. (9) estimated that environmental transmission alone may be sufficient to sustain an outbreak based on the basic reproduction number. Bravo De Rueda et al. showed that nearly 44% of transmission can result from environmental contamination with FMDV (6), while work by Colenutt et al. have highlighted the potential uses of environmental sampling for FMDV surveillance at animal aggregation points (11).

There are important biotic and abiotic factors that influence the distribution pattern and dispersal mechanisms of pathogens in the environment (27). In the case of FMD, a virus that is environmentally stable, the abiotic factors, such as temperature, RH, pH, and the matrix component (e.g., soil, feces, vegetation, inanimate surfaces, air, water, etc.) all interact to influence the duration of viral persistence. Mielke et al. (23), using a survival analysis of available literature data for FMDV persistence in the environment, showed that interactions of RH and temperature can affect survival probability. Specifically, at high RH (86%) and high temperatures (37° C), persistence (survival probability) of infectious viral particles can be expected to remain above 40% for 5 months on vegetation and 2 weeks on inanimate surfaces, suggesting that high RH provides protection for the virus when temperatures rise (23). Similarly, (7) in their review of airborne transmission of FMD noted that conditions conducive to high virus survival, such as RH >55% and gentle winds and a stable atmosphere, are needed for airborne transmission of FMD to occur (7). These effects are particularly important in settings where these conditions can be found (e.g., tropical settings, microhabitats on production sites). To understand detection patterns in these types of settings we examined a variety of environmental factors around FMD-affected herds on endemic sites.

In addition to environmental factors, how animals move within an agricultural system may influence viral dispersal, presence, and persistence. In Cameroon, there is a complex system of mobile herders (transhumant), transboundary trade herds, and sedentary (agropastoral and production) herds, which offer a variety of settings where FMD outbreaks routinely occur. The role of environmental transmission within this dynamic system is poorly understood, and virus elimination activities are not undertaken (22). This has resulted in a unique opportunity to begin to investigate patterns of environmental detection of FMD viral RNA in a natural setting.

## 2. Materials and methods

This study was conducted during 2016 on three sedentary cattle herds in the Adamawa Region of Cameroon, to characterize the spatial and temporal extent of FMD viral RNA detection around these herds. The herds were selected based on ease of access and amenability to reporting outbreaks. At the time of the study, the populations of each herd were 42, 47, and 52 head of cattle for herds 1, 2, and 3, respectively. The location and proximity to human or livestock activity varied by site with Herd 1 situated just outside a city center, Herd 2 situated in a remote location, and Herd 3 located near a city center and a local cattle market (Supplementary Figure S1). These herds are sedentary herds; however, the cattle are not cordoned in a specific site, but rather can roam within and beyond what might be considered traditional site boundaries. The three herds sell and buy animals from cattle markets and often animals from the herd accompany animals being sold. The unsold animals and those animals accompanying the herd at market are taken back to the herd. This practice could lead to the contamination of animals in those animal markets as well as introduce FMDV to the herds (21). The seroprevalence for non-structural proteins (NSP) ranges from 51.06 to 77.78% in cattle and 10.81 to 27.27 in sheep in this region (Table 1). There are two main seasons, the rainy season (April–November) and the dry season (December–March). The climate is tropical with average temperatures >20°C and < 40°C while RH ranges from ~30 to 80% (12). At each site, soil surface, air, and fomite samples were collected, and environmental conditions were measured and recorded during reported FMD outbreaks. In the endemic setting, cattle herds are infected at various times throughout the year and especially at times when new susceptible animals are present in the population (e.g., births). Cameroon is an endemic country and this study relied on the herder’s knowledge to identify when there was an outbreak of FMD in their herd. Previous infection in these herds is expected due to the endemic situation in the Adamawa Region. However, there is some use of movement restriction, antibiotics, and traditional formulations to treat suspect cases. Sample collection in each herd began at the first owner-reported outbreak based on clinical signs and then again at, +2 days, +5 days, +14 days, +21 days, and + 28 days from the first report.

**Table 1:** The NSP seroprevalence in each herd site location and on eneighboring region for cattel and sheep.

Site (Herd code)	Cattle (%)	Sheep (%)
Velambai (herd 1)	53.85	11.11
Mbidjoro (herd 2)	77.78	27.27
Soukourwo (herd 3)	77.27	14.71
Galim (neighboring herd)	51.06	10.81

The analysis was completed in two parts, the first was a descriptive analysis for air, fomite, and soil surface samples, and the second was an advanced statistical analysis using the soil surface samples only. During the project, it was determined that there was not a reliable energy source in the field to collect air samples, therefore the volume of air sampled was not calculated and this data was used as presence/absence data. Additionally, at the time of study design, there was a lack of available literature data on FMD viral RNA survival in air, and there were limited samples for fomites in the study, which reduced our ability to run further analyses on these two sample types. However, using the soil surface data, Bayes theorem (4) was applied to the soil surface data to generate a model, produce prior values, and evaluate the posterior probability distribution of the model parameters conditional upon the predictors [RH (r), temperature (t), and an interaction (t:r)] and the response (probability of detection), rather than calculating a single point estimate as done in a classical frequentist approach.

### 2.1. Data On environmental conditions

Environmental conditions, including ambient temperature, and RH were recorded three times for at least 1 min upon arrival and prior to departure at each of the three sites. Relative humidity and temperature data were collected using a portable weather tracker (Krestel® 4,500 made in United States).

### 2.2. Surface samples

#### 2.2.1. Sample collection

Soil surface sampling was completed using a 10 cm x 10 cm template to mark locations and gently swab the entire area with an electrostatic cloth. Prior to each use, the template was disinfected and dried. The disinfection method was carried out using Virkon® S, which is an effective disinfectant for surface microbes. One tablet was diluted in 500 mL of water and sprayed on the shoes of researchers, herders, and car tires. The locations of soil swabbing occurred in the center of the main cattle resting area (0 m) and at 50 m, 75 m, and 100 m points along three pathways, designated by cattle presence, and use as: (i) high cattle traffic, (ii) medium cattle traffic, and (iii) low to no cattle traffic (Supplementary Figure S2). When present, the herder, cattle owner (who does not accompany the herd in most cases), and a member of the herder’s family (not associated with cattle) had their shoes or feet sampled along with drivers, research personnel, and vehicle tires. Sampling shoes/feet and tires was completed by gently swabbing the entire bottom surface of the shoes/feet of each person using a pair of sterile forceps and a Kimwipe®. This sampling allowed for broad capture of viral RNA and movement associated with animals, human handlers, and fomites that may transfer FMD viral RNA.

#### 2.2.2. Sample storage

The electrostatic cloth, used for swabbing soil, shoes/feet, or vehicle tires was placed in a tube with 10 mL viral transport media (VTM) and shaken vigorously for 20 s. VTM consisted of 500 mL DMEM (Dulbecco’s Modified Eagle Medium) (Life Technologies, 12,430–047), 500 mL Glycerol (Sigma, G5516-1 L), and 10 mL of Antibiotic/Antimycotic (Life Technologies, 15,240,062) (mixture formulated from personal communication with Plum Island Animal Disease Center). Two labeled cryotubes were used to hold 2 mL of the sample in VTM (1 mL each). The remaining buffer, cloth, and tube were then discarded. We attempted to gather negative control samples including swabs of the researcher’s shoes and vehicle tires upon entry and exit of the site. When the research team arrived on site, they parked off-site to prepare for entry to the site. The off-site arrival location was the designated place that negative controls of the researcher’s shoes would be collected. Researcher’s shoes and vehicle tires were exposed to the ground and/or road surface near the site prior to entry sampling. One tube of VTM was filled and labeled at the field site with no sample added as a field blank. All sample tubes were stored in a cooler on ice and then taken to a − 20°C freezer on the day of collection. At the end of the sampling period, the samples were taken to the final laboratory destination and placed in a − 80°C freezer. The researchers cleaned and disinfected their vehicles, shoes, and equipment after each visit to the site with Virkon®, and the sampling equipment was thoroughly cleaned at the field laboratory with soap and water using clean ground water sources.

### 2.3. Air samples

#### 2.3.1. Sample collection

Air sampling was used to capture virus shedding from the host through aerosolized fluids or re-suspension from the surrounding environment by movement and wind disturbance. Impinger fluid was prepared with 370 mL of Glasgow Eagle’s Medium, 5 mL pen/strep, 5 mL of Fungizone or Amphotericin B, 10 mL of 5% Bovine Serum Albumin (BSA), and 10 mL of 1 M Hepes (Pirbright Institute guidance). Sampling took place during periods of no rainfall using cyclonic samplers (Microtek®) set up with 30 mL impinger fluid and a car battery as the power source. Air samplers were set up along the same traffic pathways where soil samples were collected (Supplementary Figure S2) and allowed to run for 2 h while cattle were present in the main resting area. Researchers remained on site to prevent disturbance by people or animals. In the center location an individual remained near the sampler to prevent damage to the sampler by the cattle. During operation, this person took care not to pass their hands or clothing over the sampler intake. Cyclonic samplers were operated starting at the farthest distance (100 m) and working toward the center of the herd (0 m). Each sampler was disinfected using the Virkon® S solution between sampling events according to procedures detailed by standard operating procedures personally communicated from the Torremorell laboratory at the University of Minnesota, which has worked extensively in evaluating methods of air sampling for viruses such as influenza (28).

#### 2.3.2. Sample storage (Impinger fluid)

At the end of each two-hour sampling period, reservoirs were removed, and the impinger fluid was decanted into a labeled graduated cylinder. To maintain a volume of 30 mL, impinger fluid was added, and 2 mL of impinger fluid was saved and divided into two cryotubes. All sample tubes were stored in a cooler on ice and then taken to a − 20°C freezer on the day of collection. At the end of the sampling period, the samples were taken to the final laboratory destination and placed in a − 80°C freezer.

### 2.4. Laboratory analysis: rRT-PCR

Samples were tested for the presence of FMD viral RNA by amplifying the 3D polymerase gene via rRT-PCR at the National Veterinary Laboratory (LANAVET) in Garoua, Cameroon, following protocols published elsewhere (24). The primers and probes used were in accordance with the publication by Callahan et al. (8, 24) and the cycle threshold was set to <40 for a positive result (26). Due to laboratory and funding constraints, virus isolation was not performed on positive samples.

### 2.5. Model development

Data was separated into herd, distance, and path (set as factors), and temperature, RH, and day (centered and scaled to compare across units). Pairwise plots were used to visually inspect correlation across variables, which indicated that temperature and RH are highly correlated. Using the Moran’s I test, spatial autocorrelation was tested with a distance limit of >0 or < = 0.5, meaning that points with distances below 0.5 km are related. This test indicated that no spatial autocorrelation existed, across the three sedentary herd sites in Cameroon, with a value of *p* of 0.094 (Supplementary Table S1). A Bayesian analysis using informed and non-informed priors for all predictors was used to evaluate the field data. The use of informed or non-informed priors did not alter the model and therefore the remaining analysis was completed using the non-informed priors. The model with the informed priors is in the supporting documentation (Supplementary Tables S7–S9).

### 2.6. Generalized linear mixed effects model development

The Bayesian regression analysis was completed to evaluate the effect of environmental factors on the probability of detection, the models included:

- Probability of Detection ~ (1| Herd) + V1 + V2 + V3 + V4 + RH
- Probability of Detection ~ (1| Herd) + V1 + V2 + V3 +  V4 + temperature
- Probability of Detection ~ (1| Herd) + V1 + V2 +  V3 +  V4 + RH + temperature
- Probability of Detection ~ (1| Herd) + V1 + V2 +  V3 + V4 +  RH + temperature + RH:temperature

The variables V1, V2, V3, and V4 represent the distance from the center of the herd (main resting area) at 0, 50, 75, and 100 m respectively, while ‘1|Herd’ is the random effect of herds in the study. Herds were set as a random effect to account for variability that may exist between herds in the field study based on the day of reporting, number of cattle infected, and other differences.

## 3. Results

**Figure 1:**
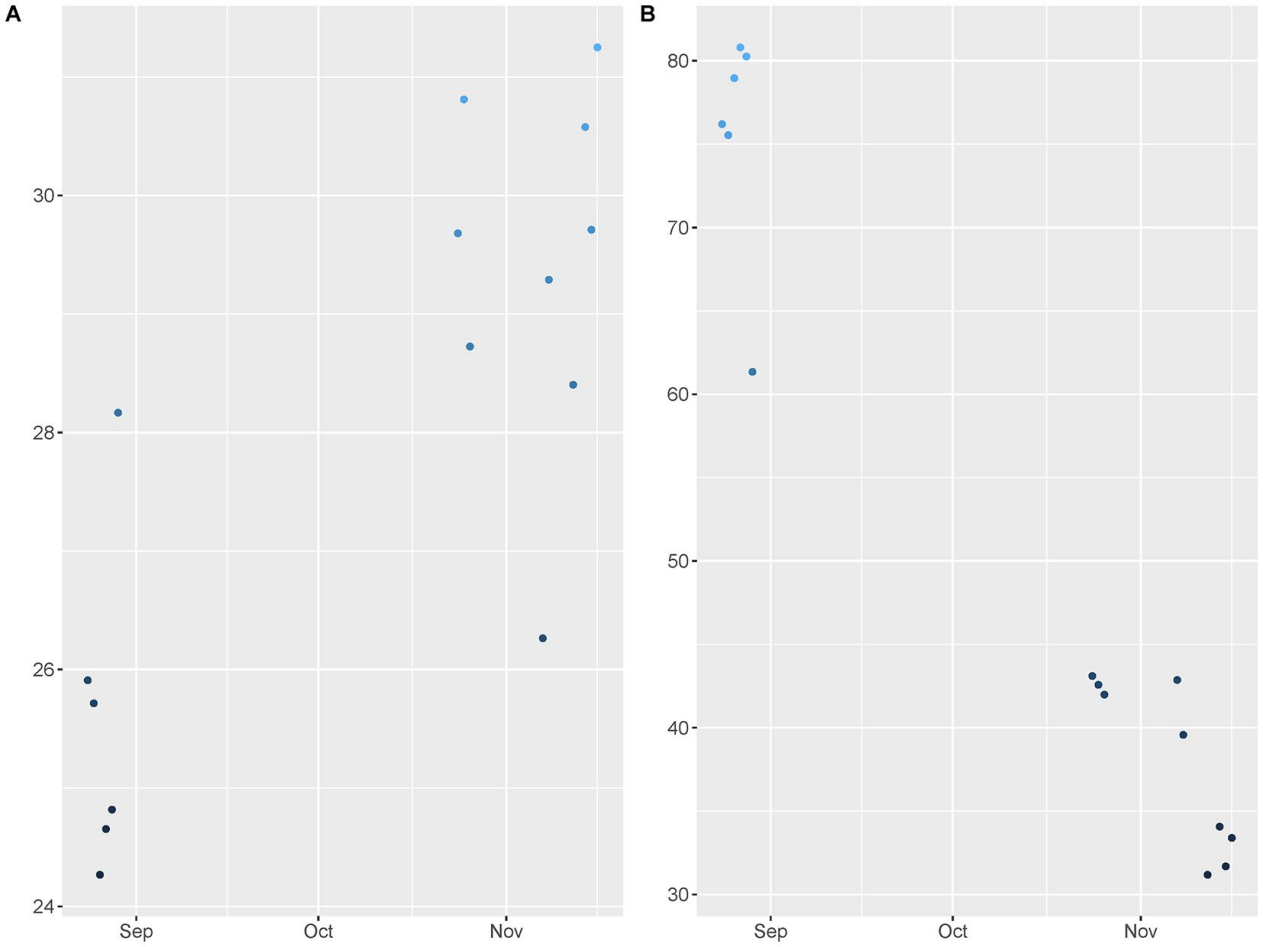
Field data points for **(A)** Temperature and **(B)** Relative humidity. The data points were collected across study activities from August to November in Cameroon West Africa.

To confirm the presence of FMD viral RNA in the environment, rRT-PCR was completed for 292 soil surface and fomite samples and 182 air samples (data is available in supporting documents). Through descriptive statistics and logistic regression, using Bayesian methods, parameters related to environmental detection of FMD viral RNA were estimated. Sampling was completed between August and November of 2016, when it is expected that temperatures increase, and RH decreases as the seasons change from wet to dry (Figures 1A,B).

### 3.1. Descriptive statistics

Using JMP (32), we analyzed the test positive proportions for FMD PCR-positives samples from fomite, soil surface, and air across the three study herds, designated as Herd 1, Herd 2, and Herd 3.

#### 3.1.1. Fomites

**Figure 2:**
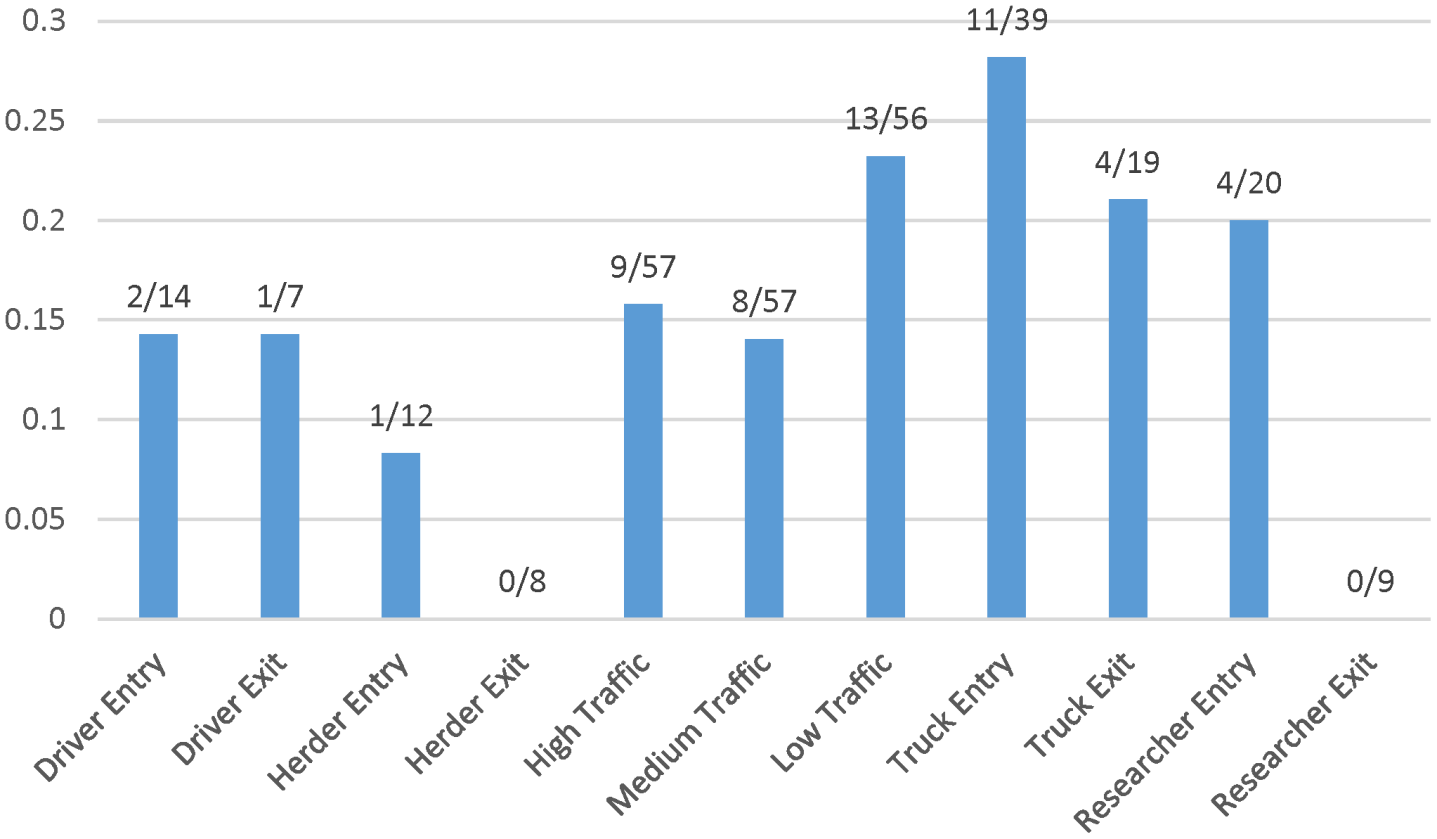
Test positive proportion of fomite and surface soil samples collected from field sites. These data points are listed by entry and exit for fomites and level of traffic for soil surfaces.

The entry and exit values indicate differences for drivers, herders, vehicles, and researchers, suggesting that fomites entering the site on researchers (20%), driver shoes (14%), and vehicle tires (28%) have a higher test positive proportion than fomites on the site herders’ feet/shoes (8%) (Figure 2). Overall, vehicles, researchers, and drivers tested positive upon entry to the sites more often than herders, who tested positive at Herd site 2 only. Exit samples tested positive for vehicles and drivers at Herd site 2 and positive for vehicles at Herd site 3, while at Herd site 1 there were no test positive results across fomites on exit. Additionally, at the exit point for Herd site 2, driver and vehicle samples had a higher test positive proportion, while for Herd site 3 vehicle samples had a higher test positive proportion compared to the entry point (Table 2). Due to low sample numbers, testing for significant differences between these fomite types was limited.

**Table 2:** The test positive proportion of fomite types at entry and exit for herd sites 2 and 3, showing that vehicles and drivers have a higher test positive proportion at exit compared to entry.

Location	Herd 2				Herd 3			
Sample	Driver	Herder	Vehicle	Researcher	Driver	Herder	Vehicle	Researcher
Entry	0.20	0.25	0.16	0.0	0.0	0.0	0.18	0.14
Exit	0.50	0.0	0.33	0.0	0.0	0.0	0.30	0.0

At herd site 1 there was zero test positive proportion at exit.

#### 3.1.2. Soil surface

**Figure 3:**
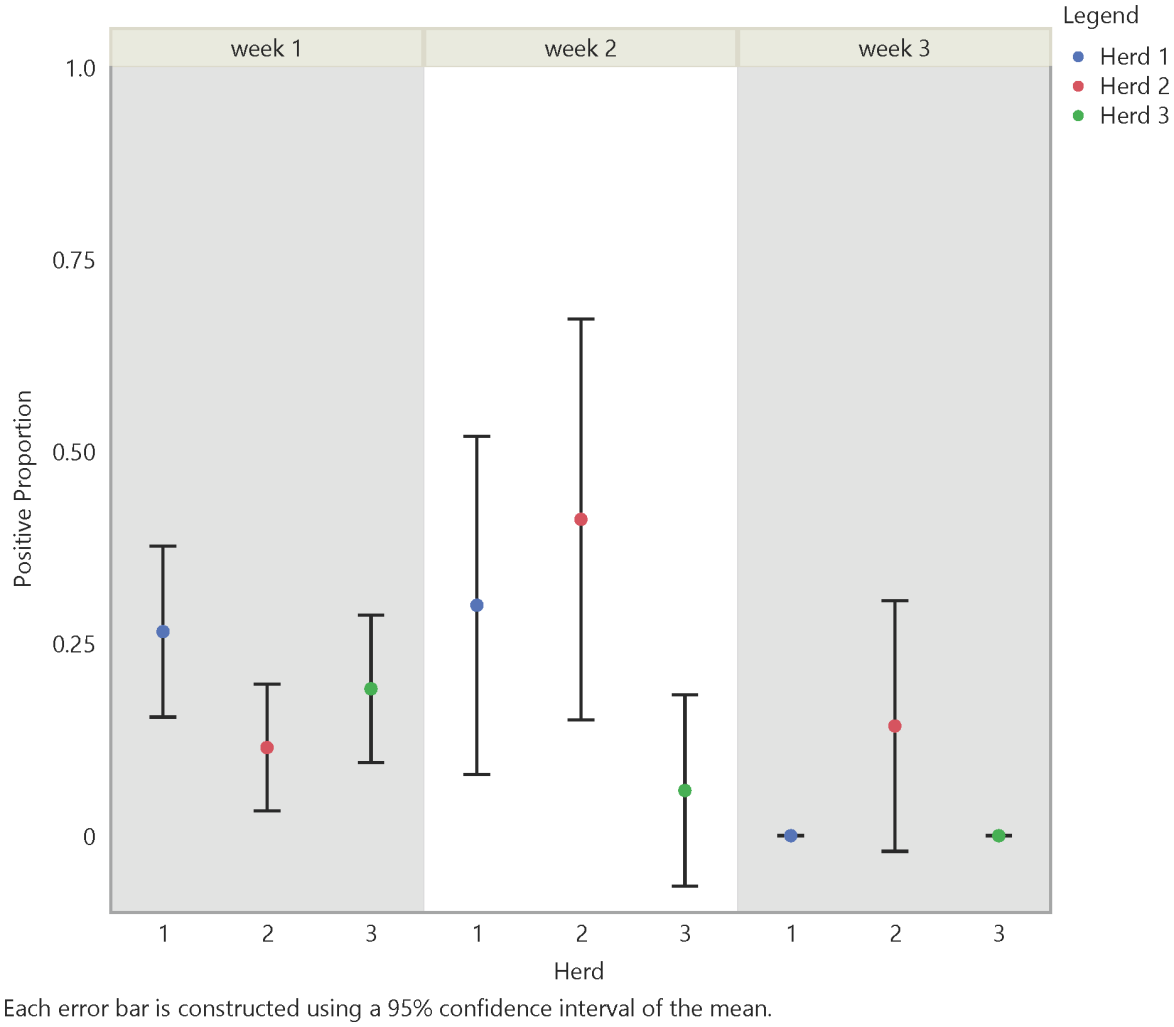
Temporal test positive proportion of soil surface samples, showing the comparison of test positive proportions by time and herd. There was a significant difference in detection between week 2 and week 3 (value of *p* = 0.0124) and between week 1 and week 3 (value of *p* = 0.0265).

**Figure 4:**
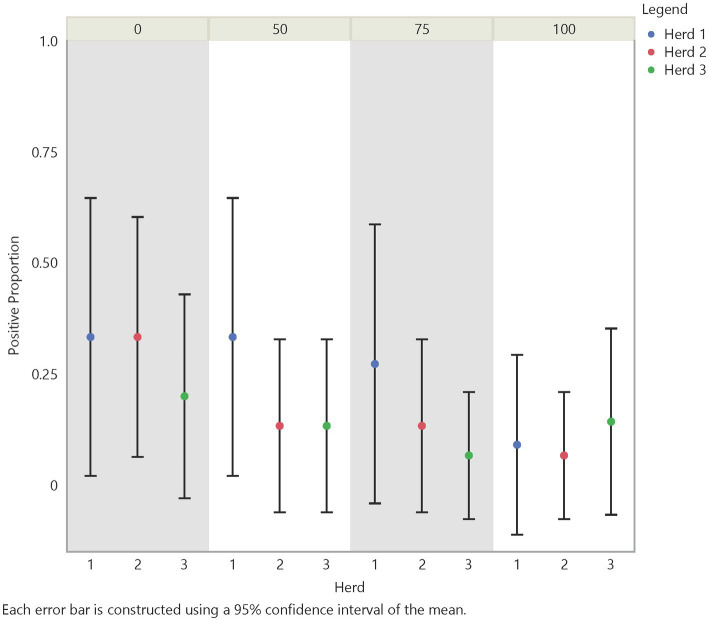
Spatial test positive proportion of soil surface samples, illustrating the test positive proportions by distance and herd. Across distance there was as significant difference between distance 0 m and distance 100 m (value of *p* = 0.0305).

Initial analysis suggests an influence on FMD viral RNA detection from time, herd, distance, and traffic pathways. The test positive proportion for traffic pathways suggests that the low/no-traffic pathway had a higher positive proportion on soil compared to the high-traffic and medium-traffic pathways with overall test positive proportions of 23, 16, and 14%, respectively (Figure 2). Using the Chi- Square test. We found no significant difference in detection probability between the pathways or the herds. However, a higher probability of FMD viral RNA test positivity was indicated for week 1 compared to week 3 and for week 2 compared to week 3, with value of ps of 0.0265 and 0.0124, respectively (Figure 3 and Supplementary Table S2). Detection of FMD viral RNA by distance was more probable at the center of the herd (0 m) than at the farthest distance (100 m), with a value of p of 0.0305 (Figure 4 and Supplementary Table S2). As this was only the first step in analysis of the soil surface data, we did not report *p*-values corrected for multiple comparisons.

#### 3.1.3. Air

**Figure 5:**
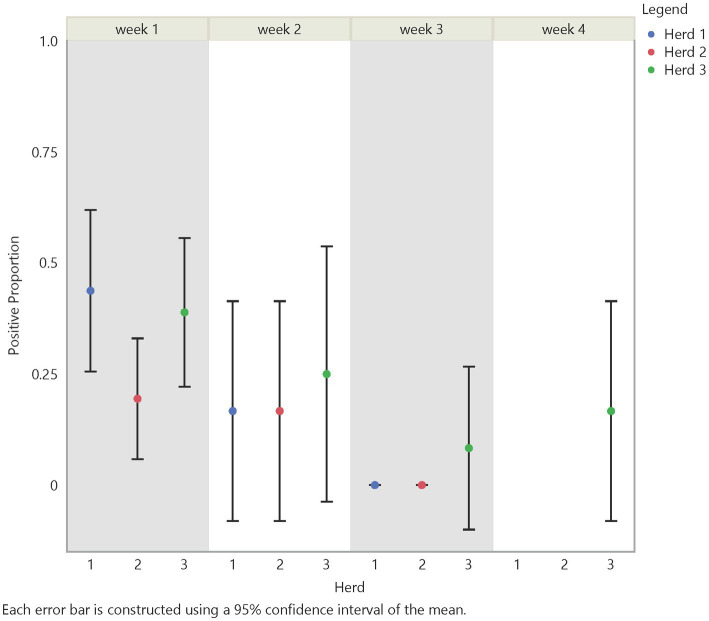
Temporal test positive proportion of air samples, which showed significant differences between week 2 and week 3 (value of *p* = 0.0177) and week 1 and week 3 (value of *p* = <0.0001).

**Figure 6:**
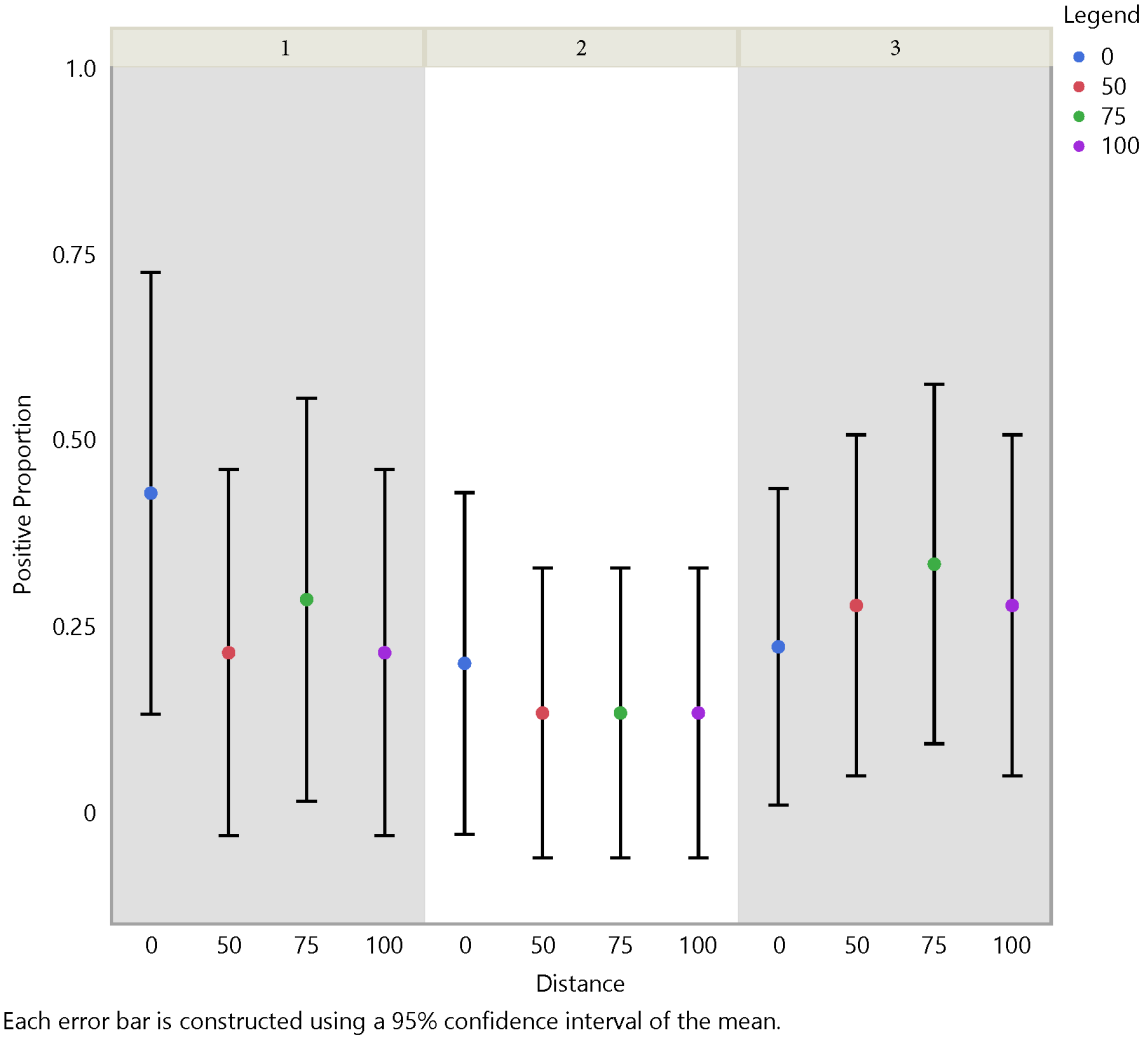
Spatial test positive proportion of air samples, which did not have significant differences in FMD viral RNA detection from the center to the periphery of the site. However, a significant difference in detection was shown between herd 1 and herd 2 (value of *p* = 0.0339).

Analysis of air samples also focused on the test positive proportion of FMD viral RNA samples across herd, time (days/weeks), distance, and traffic pathway. Similar trends in detection across time and distance with some variation between herds were noticed (Figures 5, 6). For instance, test positive proportion was 0.43 at Herd 1 compared to 0.2 at Herds 2 and 3 at distance 0 m. Additionally, Herd 3 showed an increasing trend in detection moving away from the center of the herd and reached 0.33 at 75 m before decreasing. Analysis of the traffic pathways suggested that the high-traffic pathway had higher detection within 50 m of the center with a test positive proportion of 0.33 compared to 0.26 for medium- and low-traffic pathways at the center (0 m) and 0.33 compared to 0.14 and 0.17 for medium- and low-traffic pathways at 50 m. The low- or no-traffic pathway had the same detection probability at distance 100 m compared to the center of the herd of 0.26.

Using the Chi-Square test, we made similar comparisons across time, herd, distance, and traffic pathway. We found a higher probability of detecting FMD viral RNA at Herd 1 than Herd 2 with a value of *p* of 0.0339. We did not find a difference in detection probability across distance (Figure 6) but, similar to findings in soil samples, there was a significant difference over time (Figure 5). The analysis suggests a higher probability of detection at week 1 compared to week 3 and week 2 compared to week 3, with *p*-values of <0.0001 and 0.0177, respectively (Supplementary Table S3). Figures 5, 6 illustrate the test positive proportion for the significant comparisons with probability intervals, showing higher uncertainty during weeks 2 and 4. Furthermore, there was a difference in detection probability between the study sites, with a higher probability of detection at Herd 1 compared to Herd 2. All non-significant comparisons for herds, pathways, distance, and time can be found in Supplementary Tables S4, S5 of the supporting information. As previously mentioned, the limited understanding of detecting FMD viral RNA in this environment drove us to use multiple univariate comparisons to avoid missing avenues of investigation for future work. A Bonferroni correction suggests that significance thresholds for *p*-values are 0.0083 for the effect of distance on detection in soil and time on detection in air, and 0.0167 for the effect of herd on detection in air and time on detection in soil.

### 3.2. Generalized linear mixed effect model selection using Bayesian methods

To investigate the influence of environmental factors on FMD viral RNA detection on the soil surface, we used generalized linear mixed effects models. Initially these models included day as a covariate, but this was not significant and was subsequently dropped from further analysis. Therefore, estimates may be considered as averages over the first month after reporting. When RH and temperature were modeled individually (Models I and II), they were both significant, while only temperature was significant when these variables were modeled together (Model III). Because RH exhibits variability across season in our study system, and was significant on its own, we tested an interaction term between RH and temperature and found this to be significant (Model IV). The DIC (deviance information criterion) for all models was within 5 points, but because the interaction term was significant, Model IV was chosen for further analysis. Model IV with non-informative priors was used to complete the rest of the analysis. From the model we found that RH, taken individually, did not suggest an effect on the detection probability (odds ratio of 0.99), while increasing temperature, taken individually, reduced the probability of detection (odds ratio of 0.40). However, the interaction between temperature and RH was shown to increase the probability of detection with an odds ratio of 2.19 (Table 3).

**Table 3:** The odds ratios for the variables included in the Bayesian logistic regression, Model IV.

Odds Ratio (OR) for Model IV		
Variable	OR	CI
RH	0.99	(0.40, 2.44)
Temperature	0.40	(0.15, 0.98)
RH:Temperature	2.19	(0.98, 4.86)
Distance 50 m	0.54	(0.17, 1.62)
Distance 75 m	0.39	(0.12, 1.26)
Distance 100 m	0.23	(0.06, 0.89)

Raw model output can be found in Supplementary Table S7.

For the distance covariates (V1–4) our analysis showed that the probability of detecting FMD viral RNA decreases as distance increases from the center of the herd at the main resting area on soil surface samples (Table 3 and Supplementary Table S2). Although the odds ratios decreased with increasing distance, the odds of detecting FMD viral RNA at 50 m and 75 m were not significantly different than the odds of detection at 0 m, while the odds of detecting FMD viral RNA at 100 m was significantly lower than the odds of detection at 0 m. This finding is supported by the previous univariate Chi-Square test that indicated a measurable difference in detection between distance 0 m and distance 100 m.

### 3.3. Predictions

**Figure 7:**
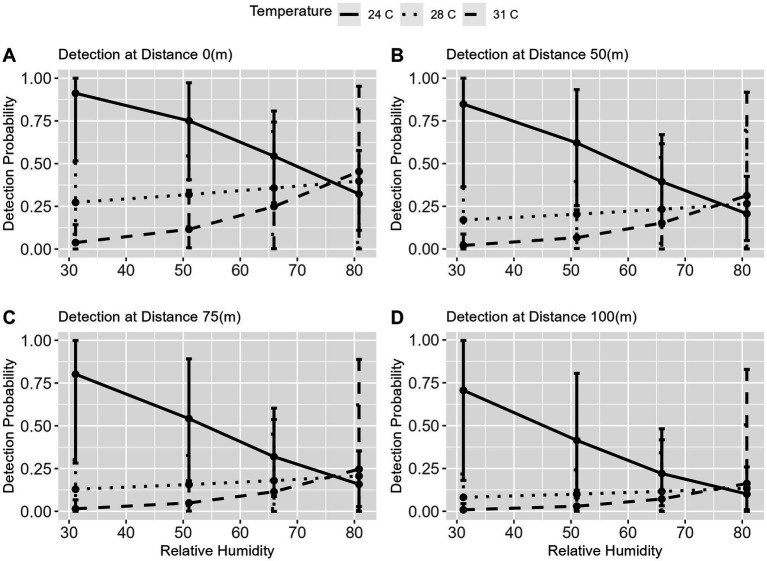
Predictions of FMDV Detection across distance based on environmental factors. This figure displays the results from the non-informed model used for prediction analysis for the detection of FMDV at each distance, 0 m **(A)**, 50 m **(B)**, 75 m **(C)**, and 100 m **(D)**. The figure shows that the overall trends are similar across the distances, with detection increasing at the highest temperature (31°C) when RH increases. This also suggests that this effect is more pronounced at the center of the herd **(A)**.

To explain the observed interaction between temperature and relative humidity in soil surface samples we used Model IV to predict FMD viral RNA detection probability over distance from the center of a herd by temperature and RH values found in our study region. These predictions show the interaction of temperature and RH, and suggest that as temperature increases, higher RH values increase detection probability (Figures 7A–D). The comparison across temperatures from the lowest (24°C) to highest (31°C) suggests that when temperatures are at 24°C the probability of detection decreases with increasing RH but as temperatures increase to 28 and 31°C the probability of detection increases with increasing RH. Across distance the same pattern is seen and the increase in detection is most pronounced at distance 0 m, with a detection probability of near 0.45 at 31°C and 85% RH. This relationship was previously alluded to in the Chi-Square comparison of soil surface samples (Supplementary Table S2), which suggested a difference in detection between distance 0 m and 100 m.

## 4. Discussion

As previously stated, FMD viral RNA has been shown to be environmentally stable under optimal conditions, and FMD remains a disease of high priority globally. Endemic FMD poses a continued threat to livestock health and production and local livelihoods, and requires that local governments and livestock producers continue to plan for the possibility of FMDV transmission into their agricultural communities (15–17, 34, 38). The on-going burden of FMD can be mitigated by improving our understanding of environmental transmission, where FMD viral RNA may be detected, and the implications for virus elimination strategies as part of a disease control program in endemic, epidemic, and FMD free regions (30). In this study we attempted to understand various environmental drivers impacting viral detection in production settings in an endemic region with natural infection and no mitigation strategies (including no vaccination), which can serve as a baseline for further work.

The environmental conditions in this region fluctuate between a rainy and dry season with RH being highest from April to November (rainy season), and peaking near 80% between July and September, while temperatures fluctuate between 20 – <40° C throughout the year (18). Similar variations in environmental conditions can be found in regions of the United States and other countries around the world, which allows for comparisons of the patterns of FMD viral RNA detection in the study site to other areas for outbreak preparedness and response planning. Although, consideration for the study location, time period of sampling, and production style (cattle movement and mitigation activities), should be taken into account, this data can provide details about locations within livestock production sites where FMD viral RNA detection *via* environmental sampling is likely to occur. For instance, we found that an interaction of RH and temperature resulted in an increase in detection probability when these environmental factors increased together. This important finding suggests that as RH increases at high temperatures (>28° C) FMD viral RNA detection probability increases, reaching 45% probability of detection on the soil surface in the immediate area of a cattle herd, 31% probability of detection at 50 meters from the center, 25% probability of detection at 75 meters from the center and just under 20% probability of detection at the periphery (100 m) (Figures 7A–D). This finding, in particular, is supported by our previous survival analysis of literature data, which predicted increased persistence under extreme temperatures (37° C) and high RH (86%) (23). The review of FMDV aerosol spread by Colenutt et al. (11), also noted the impacts of relative humidity on the stability of the virus in aerosols, while noting that this varied by FMD serotype, with serotype A viruses more stable than serotypes O and C under comparable relative humidities. While this study did not identify virus serotypes, expectations around environmental sampling outcomes may need to be tailored to account for specific serotype differences, particularly if environmental sampling is being used as a component of a surveillance system.

These findings can influence how we think about using environmental sampling to delineate patterns of risk and exposure during FMD outbreaks, particularly in settings where high temperatures can be accompanied by high RH. These settings can include both regional and microclimate conditions, including areas within a production site, which could be targeted for cleaning and disinfection treatment or other virus elimination strategies when the right conditions are met. Managing virus in the environment is only one part of a control strategy and would still need to be combined with biosecurity and management of infected and convalescent animals. The environmental sampling results in this study pose questions as to the level of contamination around an outbreak site and the origin of this contamination. Multiple sources of FMD viral RNA particles could exist, including external sources to the herd such as human and animal movements associated with livestock markets and roadways. Our results suggest a difference in detection from the center out to the periphery of the site. This spatial difference, with higher detection probability at the center of the herd compared to the periphery (100 m), was indicated in the soil surface swabs only; air sampling did not have a significant spatial difference. This introduces questions about the mechanism of dispersal. Does increased RH and temperature cause aerosols to remain aloft, or are aerosolized FMD viral RNA particles being transported from the center of the herd beyond the distance fomites are being transported? The latter is quite plausible because FMD viral airborne spread has the potential to travel 0.1 km (or 100 m) from as few as 10 infected cattle ((13)) under ideal conditions. (7) noted that aerosols from other sources such as skin and fomites can be re-aerosolized through husbandry practices including the movement of people or animals, which may amplify the locations and distances at which FMD viral RNA can be found.

Additionally, the findings raise questions about the role of neighboring sources of FMDV and how they may impact detections on surrounding farms. The phenomenon of local area spread has been routinely used in FMD modeling to represent spread by unknown causes or mechanisms that are difficult to trace (e.g., wildlife or rodents) over short distances, which could be linked to environmental spread and contamination with the virus (31). In our study we found that our negative controls (researcher’s shoes on entry) tested positive at herd sites 1 and 2 but based on study standards for cleaning and disinfecting shoes and vehicles, we believe that the researchers were not a source of contamination but may have encountered a contaminated environment prior to entering the study site. In a recent study, environmental surveillance methods were assessed by sampling areas where FMD outbreaks were either ongoing or had occurred within the past 4 weeks, and investigators also recovered FMD viral RNA > 28 days post outbreak (10). As suggested in Colenutt et al., environmental detection of FMD viral RNA after outbreaks provides a basis for non – invasive surveillance and monitoring mechanisms in endemic areas (10, 11). We can take that a step further to help inform and understand the potential extent of the affected area and the timing for reliance on elimination through natural degradation, and cleaning and disinfection under environmental conditions at the outbreak location, which present a more feasible approach than testing. Many approaches use long periods of fallowing and have well-documented histories of no transmission once animals were returned to the environment (35–37).

Limitations in this study include the small number of outbreaks sampled, limited time period of sampling, and limited fomite sampling. Although, beyond the scope of this study, the use of virus isolation to understand when infectious virus is present as well as genetic sequencing to understand virus diversity in the environment would be beneficial to improve our understanding of the nuances of environmental contamination and detection. Bolstering the sampling effort with an increase in the number of outbreak sites and including non-outbreak sites where detection of FMD viral RNA is expected to be zero (non-livestock areas) would better elucidate the pattern of environmental presence of FMD viral RNA in this setting. As a start, our work suggests that this is an interesting area of research which may have more impact on FMD transmission than previously suspected. In this setting, animals were not removed from the outbreak herd site, therefore shedding could have occurred across the sampling time frame at varying levels as FMD spread through the herd. Recent work by Brown et al. (7), could be used to strengthen the sampling design. In addition, sampling other livestock types (swine, small ruminants, etc.) and testing for infectious virus from air and soil surface samples over time would greatly increase our understanding of temporal and spatial aspects of virus transmission and contamination associated with the environment and the benefits of different elimination standards [cleaning and disinfection, fallowing, or degradation by environmental factors (high temperatures combined with low RH)]. Given what we learned through this study, we would also suggest several improvements to the study design to better understand when and how researchers’ equipment may become contaminated. For instance, sampling after disinfecting equipment used on site, in addition to the entry and exit sampling completed in this study, would be helpful. Furthermore, it would be useful to clearly document through sampling that all cleaning and disinfecting procedures were followed.

Despite these limitations, we have compiled data that represents a unique set of foundational knowledge not yet captured in this region and have improved our understanding about the potential spatial and temporal patterns of FMD viral RNA detection in a natural setting. This knowledge provides new avenues of inquiry regarding outbreak response planning in free, epidemic, and endemic regions as to where and when PCR detection of environmental samples can be expected around infected cattle herds. This can be used to help target biosecurity measures, cleaning and disinfection, and improve interpretation of PCR-based test results where environmental presence of RNA may exist.

## 5. Conclusion

Our study detected widespread viral RNA positive samples in both soil and air around outbreak sites, highlighting the possibility of cross-contamination from surrounding livestock operations or structures, as well as greatly increased dispersal of viral RNA from the herd of origin. We have shown that an interaction between temperature and RH influences the spatial and temporal detection of FMD viral RNA in the environment, which provides a foundation for understanding patterns of FMD viral RNA detection that can inform mitigation strategies in endemic, epidemic, and free areas.

## Data availability statement

The original contributions presented in the study are included in the article/Supplementary material, further inquiries can be directed to the corresponding author.

## Ethics statement

The animal study was reviewed and approved by the Ohio State University Institutional Animal Care and Use Committee; USDA; School of Veterinary Medicine and Sciences at the University of Ngaoundere (Department of Parasitology and Parasitic Disease guidelines). Written informed consent for participation was not obtained from the owners because a verbal consent was acquired from the cattle owners prior to swab collection.

## Author contributions

SM completed the literature review, data cleaning and analysis, and wrote the manuscript. SL conducted the field work, sample processing, and review and editing of the manuscript. RG provided the study design conception, analysis guidance, and review and editing of the manuscript. AD provided the study design conception and review and editing of manuscript. SD provided the study design conception, field and sample processing oversight, and review of the manuscript. MA provided the study design conception, field and sample oversight, and review of the manuscript. All authors contributed to the article and approved the submitted version.

## Funding

This material was made possible, in part, by a Cooperative Agreement from the United States Department of Agriculture’s Animal and Plant Health Inspection Service (APHIS). It may not necessarily express APHIS’ views. Additionally, this work was funded by NSF grant DEB-1015908 as part of the joint NSF-NIH Ecology of Infectious Disease program.

## Conflict of interest

The authors declare that the research was conducted in the absence of any commercial or financial relationships that could be construed as a potential conflict of interest.

## Publisher’s note

All claims expressed in this article are solely those of the authors and do not necessarily represent those of their affiliated organizations, or those of the publisher, the editors and the reviewers. Any product that may be evaluated in this article, or claim that may be made by its manufacturer, is not guaranteed or endorsed by the publisher.
