# Supplementary material for: Patterns of foot-and-mouth disease virus detection in environmental samples in an endemic setting
Source: Front Vet Sci. 2023 Jun 16;10:1157538. doi: 10.3389/fvets.2023.1157538 (PMC10312077; doi:10.3389/fvets.2023.1157538)
Supplement: Supplementary file 5 [file Table_1.DOCX]

Supplementary Materials

Detection of Foot-and-Mouth Disease Virus in Environmental Samples on Cattle Herd Sites in Cameroon

Sarah R. Mielke^*a,g^, Lendzele, Sevidzem^b^, Delgado, Amy H.^c^, Abdoulmoumini, Mamoudou^d^, Dickmu, Simon^e,f^, Garabed, Rebecca^a^

^*^Correspondence: Sarah.mielke@usda.gov

**Morans I:**

The gps coordinates were reported in DD° MM.MMM (Degrees and Decimal minutes) format from the gps recorder. I converted these to decimal degrees by the following formula:

Decimal Degrees = (DD° + (MM/60) + ((.MMM/60)/3600)), then the decimal degrees are used in the R code for location on maps.

Location was used in Morans I test to determine if spatial autocorrelation is present in the variable FMDV (present/absent) at increasing distances used as the measure for connectedness. When I use the distance limit of 0.25, meaning that points at a finer scale are connected, the Morans I p-value is significant at 0.038. When the distance value is increased to 0.50 and 0.75, indicating a coarser spatial scale, the Morans I p-value is non-significant at 0.094. Using the distance limit of 0.50 I fail to reject the H_o_, which states that there is zero spatial autocorrelation among the points. This test indicates that at the finest scale, within the sampling site, there is spatial autocorrelation. However, because there was only one GPS coordinate for each site we assessed the spatial autocorrelation between herd study sites at a spatial scale of 0.50.

*Code*

**svtm.dists.bin <- (svtm.dists > 0 & svtm.dists <= .75)** ##tested 0.5, 0.25, 0.75

**Moran.I(svtm$fmdv, svtm.dists.bin)** testing for spatial autocorrelation based on binary distance with those >0 or <= 0.75, >0 or <=0.50, and >0 or <=0.25 are connected H_o_: There is zero spatial autocorrelation present in the variable

Table S1: Moran's I test of spatial autocorrelation among the points across three sedentary herds in Cameroon with distances set to >0 <= 0.75, >0 <=0.50, and >0<=0.25.

| Observed | Expected | sd | p.value |  |
| --- | --- | --- | --- | --- |
| -0.007034805 | -0.003436426 | 0.002149656 | 0.09414389 | >0 or <=0.75 |
| -0.007034805 | -0.003436426 | 0.002149656 | 0.09414389 | >0 or <=0.50 |
| -0.01406571 | -0.003436426 | 0.004816983 | 0.02734019 | >0 or <=0.25 |

Table S2: Significant comparisons of test positive proportion for FMDV in soil surface samples for distance and time. All non-significant comparison can be found in table S4

| Soil surface Data Comparison | Stratified | Chi Square Likelihood | p-value |
| --- | --- | --- | --- |
| Distance | Herd | 5.167 | 0.1600 |
| Distance (0:100) | Herd | 4.683 | 0.0305* |
| Time | Herd | 6.308 | 0.0427* |
| Time (week 2: week 3) | Herd | 6.254 | 0.0124* |
| Time (week 1: week 3) | Herd | 4.921 | 0.0265* |

Table S3: Significant comparisons of FMDV detection over distance, time, and herd in air samples at study sites in Cameroon. All non-significant comparisons can be found in table S5

| Air Data Comparison | Stratified | Chi Square Likelihood | p-value |
| --- | --- | --- | --- |
| Herd (1:2) | Distance | 4.499 | 0.0339* |
| Time | Herd | 18.657 | 0.0003* |
| Time (week2: week3) | Herd | 5.625 | 0.0177* |
| Time (week1: week3) | Herd | 17.622 | <0.0001* |

Table S4: Non-significant comparison of test positive proportion for FMDV detection in soil samples by herd, traffic pathway, distance and time (weeks).

| Soil Data Comparison | Stratified | Chi Square test | p-value | Chi Square Pearson | p-value |
| --- | --- | --- | --- | --- | --- |
| Herd | Path | 2.76 | 0.2514 | 2.87 | 0.2378 |
| Path | Time | 1.669 | 0.4341 | 1.711 | 0.4250 |
| Herd | Distance | 2.76 | 0.2514 | 2.87 | 0.2378 |
| Herd (LH:HBS) | Distance | 0.224 | 0.6359 | 0.224 | 0.6362 |
| Herd (Djaoro:LH) | Distance | 2.615 | 0.1059 | 2.631 | 0.1048 |
| Herd (Djaoro:HBS) | Distance | 1.394 | 0.2377 | 1.405 | 0.2359 |
| Distance(0:75) | Herd | 2.412 | 0.1204 | 2.373 | 0.1235 |

continued

Table S4 Continued

| Soil Data Comparison | Stratified | Chi Square test | p-value | Chi Square Pearson | p-value |
| --- | --- | --- | --- | --- | --- |
| Distance(0:50) | Herd | 1.056 | 0.3042 | 1.050 | 0.3055 |
| Distance(50:75) | Herd | 0.289 | 0.5908 | 0.288 | 0.5914 |
| Distance(50:100) | Herd | 1.368 | 0.2422 | 1.342 | 0.2466 |
| Distance(75:100) | Herd | 0.404 | 0.5248 | 0.402 | 0.5262 |
| Time  (week 1:week 2) | Herd | 0.952 | 0.3291 | 0.987 | 0.3204 |

Table S5: Non-significant comparisons using the Chi Square test for air samples across time, distance, traffic pathway, and herd.

| Air Data Comparison | Stratified | Chi Square test | p-value | Chi Square Pearson | p-value |
| --- | --- | --- | --- | --- | --- |
| Traffic Pathway | Herd | 0.188 | 0.9103 | 0.189 | 0.9097 |
| Traffic Pathway (MT:NT) | Herd | 0.008 | 0.9277 | 0.008 | 0.9277 |
| Traffic Pathway (HT:NT) | Herd | 0.100 | 0.7524 | 0.099 | 0.7524 |
| Traffic Pathway (HT:MT) | Herd | 0.368 | 0.5440 | 0.369 | 0.5437 |
| Herd | Distance | 5.091 | 0.0784 | 4.831 | 0.0893 |
| Herd (HBS:LH) | Distance | 3.196 | 0.0738 | 3.117 | 0.0775 |
| Herd (Djaoro:LH) | Distance | 0.252 | 0.6158 | 0.253 | 0.6150 |
| Distance | Herd | 0.948 | 0.8138 | 0.951 | 0.8131 |
| Distance (75:100) | Herd | 0.302 | 0.5829 | 0.301 | 0.5831 |
| Distance (0:100) | Herd | 0.617 | 0.4322 | 0.616 | 0.4327 |
| Distance (0:50) | Herd | 0.617 | 0.4322 | 0.616 | 0.4327 |
| Distance (50:75) | Herd | 0.302 | 0.5829 | 0.301 | 0.5831 |
| Distance (0:75) | Herd | 0.055 | 0.8139 | 0.055 | 0.8139 |
| Distance (50:100) | Herd | 0.0 | 1.0 | 0.0 | 1.0 |

continued

Table S5 Continued

| Air Data Comparison | Stratified | Chi Square test | p-value | Chi Square Pearson | p-value |
| --- | --- | --- | --- | --- | --- |
| Time (week3:week4) | Herd | 2.492 | 0.1145 | 2.963 | 0.0852 |
| Time (week2:week4) | Herd | 0.047 | 0.8292 | 0.046 | 0.8309 |
| Time (week1:week2) | Herd | 2.722 | 0.0990 | 2.571 | 0.1088 |
| Time (week1:week4) | Herd | 1.587 | 0.2078 | 1.429 | 0.2319 |

Table S6: The model output from Model IV with non-informative priors. The model DIC = 154.9.

| Model IV with weakly informative priors on all variable (N~(0, 1e-6)) | | | | | |
| --- | --- | --- | --- | --- | --- |
| **Variable** | **Median** | **Credible Interval** | **Mean** | **SD** | **psrf** |
| intercept | -0.47195 | (-1.51, 0.58) | -0.47821 | 0.57289 | 1.0003 |
| Distance 0 | 0 | 0 | 0 | 0 | -- |
| Distance 50 | -0.60552 | (-1.70, 0.52) | -0.60911 | 0.56563 | 0.99998 |
| Distance 75 | -0.92957 | (-2.15, 0.23) | -0.94276 | 0.60232 | 1.0003 |
| Distance 100 | -1.4314 | (-2.85, -0.16) | -1.462 | 0.68539 | 1.0001 |
| RH | -0.0001 | (-0.91, 0.92) | -0.0058555 | 0.46699 | 1 |
| Temperature | -0.90874 | (-1.85, 0.01) | -0.91734 | 0.47476 | 1.0003 |
| RH:Temperature | 0.7873 | (0.01, 1.60) | 0.79215 | 0.40397 | 1 |
| Herd (random) | 52.996 | (0.001, 967.25) | 211.27 | 413.83 | 1.0001 |
| deviance | 146.82 | (141.23, 155.57) | 147.5 | 3.964 | 1.0002 |
| resid.sum.sq | 23.397 | (22.50, 24.77) | 23.516 | 0.63489 | 1.0001 |

**Prior Development**

To use an informed prior in the model we used the data from our previous meta-analysis and creating prior values for the two variables temperature and relative humidity. The prior values and model output can be found in tables (S6, S7, & S8) below.

1. using the data points for *vegetation/food* to represent persistence of FMD viral RNA on the soil surface. These data points best represent soil surface data points from our field study because they come from experiments that used materials such as hay and pasture plants, which are similar to the types of vegetation that would be found on traffic pathways in the study.
2. calculating predictions from the *vegetation/food* dataset at the range of temperature (22, 24, 26, 28, 30°C) and RH (43.0, 66.0, 70.0, 86.1%) values found in the field study. The values for temperature and RH were set as the ‘newdata’ argument in a ‘survfit’ prediction and the probability of survival, at each time-point and covariate combination, was collected to create a simulated dataset of survival, translated to a binary outcome (detection), at a cut-off of 50, 75, and 90%.
3. centering and scaling the variables of temperature, RH, and day of the simulated dataset, by using the centering and scaling values derived from the field data, collected in Cameroon. This ensured that all scaling was the same for these variables across datasets.
4. subsetting the new simulated dataset to include a prediction for every day that matched a detection day represented in the field samples (days: 1.5, 4, 14, 17, 20, 21, 29, 30); because there was no reference for day 1 the reference of day 1.5 was used.
5. using frequentist methods to conduct a logistic regression model using the simulated dataset to find and collect prior estimates for the regression coefficients. The regression model, used for prior determination, incorporated a binomial distribution with a logit link, and is shown below:

$survival probabilty at 75\% \sim day+r+t$ + *t:r*Where, `t` represents temperature, `r` represent relative humidity, `t:r` is an interaction term, and day is the day since the outbreak was first reported. The cut-off value for the probability of survival was evaluated at 50, 75, and 90% and the coefficients for temperature, RH, and day were all significant when a 75% probability of survival was used. Additionally, survival of >=75% is conservative, assuming that >=75% of the original virus deposited would need to be present for us to detect it in our field sampling. Table I lists the resulting mean and variance (converted to precision (Tau (τ))) for the coefficients related to temperature, RH, and the interaction term that were pulled from the logistic regression and set as informative priors, while non-informative priors were used for the remaining variables N~(0, 1e-6) in a Bayesian regression analysis, using the ‘runjags’ package (Denwood, 2016) with JAGS and R (RStudio Team, 2016). The full RStudio code can be found in supporting information.

1. comparing the priors developed above to non-informed priors across all predictors in the final model.

Table S7: The odds ratios for the variables included in the Bayesian logistic regression, Model IV. Raw model output can be found in supporting information, table S9).

| Odds Ratio (OR) for Model IV | | |
| --- | --- | --- |
| **Variable** | **OR** | **CI** |
| RH | 0.99 | (0.40, 2.44) |
| Temperature | 0.40 | (0.16, 1.0) |
| RH:Temperature | 2.21 | (1.01, 4.87) |
| Distance 50m | 0.54 | (0.18, 1.60) |
| Distance 75m | 0.39 | (0.11, 1.22) |
| Distance 100m | 0.23 | (0.06, 0.86) |

Table S8: The prior values developed from the survival dataset to use in the Bayesian logistic regression analysis.

| Priors for GLM | | |
| --- | --- | --- |
| **Variable** | **Mean** | **Tau (τ)** |
| RH | 7.5 | 0.0019 |
| Temperature | -1.5 | 0.0203 |
| RH:Temp Interaction | 0.303 | 0.316 |

Table S9: The model output from Model IV with informative priors on RH, temperature, and the interaction term and non-informative priors on remaining variables (N~(0, 1e-6)). The model DIC = 154.9.

| Model IV with informative priors on RH and temperature (Table 4 article) | | | | | |
| --- | --- | --- | --- | --- | --- |
| **Variable** | **Median** | **Credible Interval** | **Mean** | **SD** | **psrf** |
| intercept | -0.47317 | (-1.47, 0.57) | -0.47579 | 0.58219 | 1.0041 |
| Distance 0 | 0 | 0 | 0 | 0 | -- |
| Distance 50 | -0.61089 | (-1.72, 0.46) | -0.61495 | 0.56112 | 1.0 |
| Distance 75 | -0.92728 | (-2.14, 0.20) | -0.93871 | 0.60187 | 1.0001 |
| Distance 100 | -1.4342 | (-2.82, -0.14) | -1.4579 | 0.68608 | 1.0001 |
| RH | -0.00745 | (-0.90, 0.89) | -0.0105 | 0.45915 | 1.0002 |

continued

Table S9 continued

| Model IV with informative priors on RH and temperature (Table 4 article) | | | | | |
| --- | --- | --- | --- | --- | --- |
| Temperature | -0.91165 | (-1.84, -0.009) | -0.91719 | 0.46975 | 1.0001 |
| RH:Temperature | 0.78611 | (0.0103, 1.58) | 0.79085 | 0.40097 | 1.0006 |
| Herd (random) | 56.973 | (0.001, 984.69) | 214.83 | 417.88 | 1.0 |
| deviance | 146.79 | (141.24, 155.37) | 147.45 | 3.9348 | 1.0 |
| resid.sum.sq | 23.396 | (22.50, 24.74) | 23.51 | 0.63078 | 1.0 |

**Figures**

Map of study herds in the different villages and NSP seroprevalence


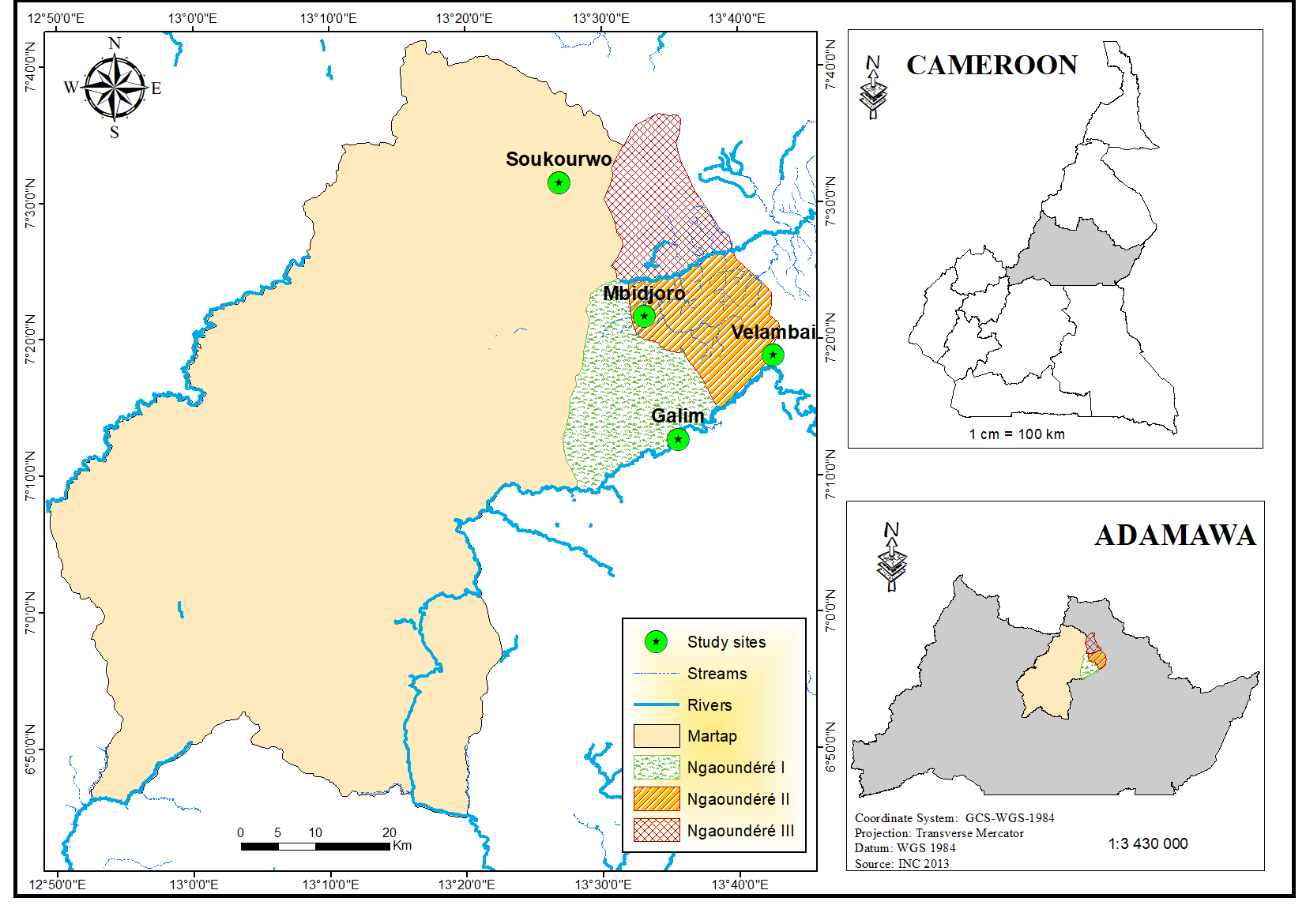


Figure S1: The map of the study region with herd sites anda neighboring site in the Adamawa region.

**
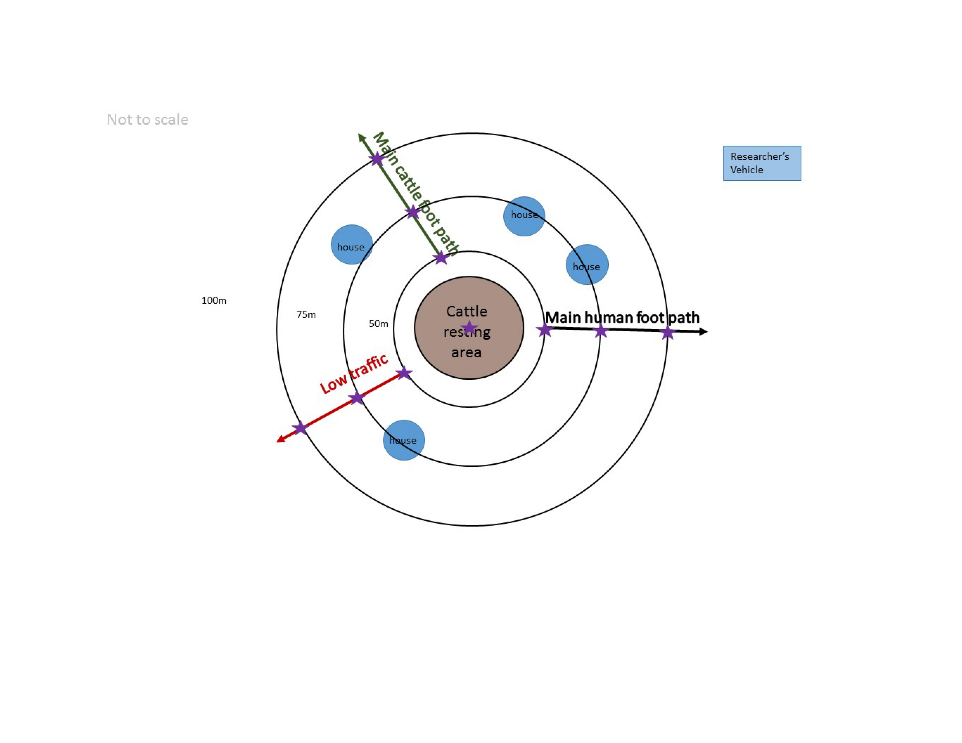
**

Figure S2: The sampling design illustrating the sampling points from the center of a herd for both soil and air sampling approaches.

**
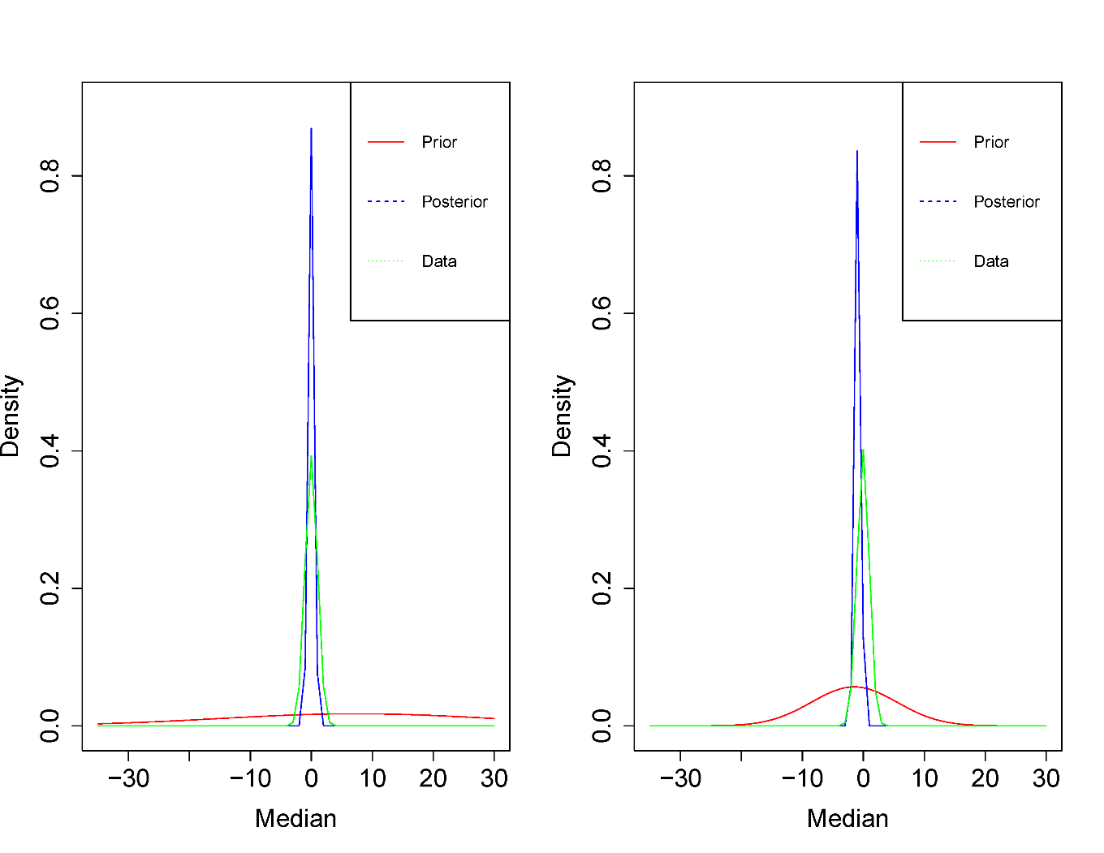
**

Figure S3: Coefficients for (a) Relative Humidity and (b) Temperature. These figures are useful for understanding how the prior values influence the posterior distribution density of the coefficients compared to the density distribution of the coefficients from the field data. Because we use priors for the RH coefficient (a) and the temperature coefficient (b) both variables must be evaluated for their influence on the posterior distribution (model outcome).

**rRT-PCR Protocol** (Laboratories & Center, 2017)**:**

Extraction of the viral RNA was completed using the Rneasy Protect Mini Kit (cat.nos. 74124 and 74126, QIAGEN ®, 2011). To begin, samples were defrosted at room temperature prior to homogenization with the vortex. After homogenizing, 140 μl of each sample was taken and deposited into labelled Eppendorf tubes and 350 μl of cell membrane lysis medium (RLT) was added to each Eppendorf tube. The sample was again homogenized using the vortex and upon completion 140 μl of 70% ethanol was added to the contents of each tube. After addition of the ethanol, 630 μl of the contents of each tube were transferred to a new collection tube containing filter columns and labeled according to the previous labels on the Eppendorf tubes and centrifuged. Centrifugation was performed four times at 8000 rpm for one minute with filter columns removed, collection tubes emptied, and filter columns replaced between each centrifugation procedure.

For the second centrifugation, 700 ul of the buffer RW1 was added and for centrifugation step 3 and 4, 500 μl of the RPE buffer was added. After the fourth centrifugation, the filter columns were again removed, collection tubes emptied, and the filter columns returned to the collection tubes. The fifth centrifugation step was completed at 13000 rpm for one minute without adding anything. Upon completion of the five centrifugation steps the filter columns were removed, and placed in new numbered Eppendorf tubes with the original label. We added 45 μl of Rnase-free water to the new Eppendorf tubes and performed a final centrifugation at 8000 rpm for one minute. Once the final centrifugation step was complete, the filter columns were removed and discarded and the Eppendorf tubes, containing the extracted RNA, were resealed immediately, placed in crushed ice, and put in the freezer at -80 ° C for storage until amplification.

**Amplification of RNA and results interpretation**

*Primers/Probes*

Forward primers (Rasmussen): 5’GACAAAGGTTTTGTTCTTGGTCA-3’;

Reverse primers (Rasmussen): 5’TGCGAGTCCTGCCACGGA-3’;

Fluorescent probe (Callahan): 6FAM-TCCTTTG CACGCCGTGGGAC-Tamra

*Amplification*
 To begin amplification 22.5 μL of Master mix was dispensed into each well of the microplate, and 2.5 µl of sample was added to each well. The microtiter plate was covered with an adhesive film and placed in the ABI 7000 thermal cycler.

The thermal cycler settings (relating to the temperature, duration and number of amplification cycles) were as follows:

- 50° C for 10 min for reverse transcription of the viral RNA (one cycle);
- 95° C for 10 min for inactivation of reverse transcription of viral RNA (one cycle);
- 95° C for 15 seconds for the denaturation of the cDNA (45 cycles);
- 60° C for 60 seconds for hybridization and elongation (45 cycles).

At the end of the elongation step of each cycle, which is one minute at 60° C, fluorescence is read. The "Cycle Threshold" is defined as the cycle number for which the fluorescence intensity intersects the baseline or "threshold"; the device software automatically sets this baseline.

*Validation criteria*

Test is validated if:

- Negative controls (extraction and amplification) all have an indeterminate CT value marked "Unknown" and below the threshold;
- Positive control extraction must have a CT value between 37-40;
- Positive amplification control must have a CT value between 16-19

*Results interpretation*

- Samples will be considered positive if their curves rise above the threshold before the 40th cycle (Paprocka et al., 2010).
- Samples with a CT value between 40-45 are considered doubtful and the test must be reviewed to define the status of the sample, in relation to the infection.

RStudio Code

CODE

##Environmental Persistence Analysis--Cameroon##

##########################

library(readxl)

svtm <- read_excel("svtm.xlsx")

##look at data##

#View(svtm)

##Packages for this analysis##

library(runjags)

library(rjags)

########## Data Prep #########################################

sapply(svtm, function(x) length(unique(x))) ###displays the number of unique values in each column

##Scale and center, and set data as factors as needed##

svtm$ID<- as.factor(svtm$ID)

svtm$Sample<- as.factor(svtm$Sample)

svtm$Herd<- as.factor(svtm$Herd)

svtm$date<- as.factor(svtm$date)

svtm$temp<- scale(svtm$Avetemp, center = TRUE,scale = TRUE) #scale so that each variable is comparable across units, have to reverse scale to interpret

svtm$rh<- scale(svtm$AveRH, center = TRUE,scale = TRUE) ##using the values from here that can be found in drop down attributes

svtm$dy<- scale(svtm$day, center = TRUE, scale = TRUE) ###as the values to put in the prd data for center and scale

svtm$ds<-as.factor(svtm$dist)

svtm$pth<- as.factor(svtm$path)

####################################################################

## Methods for prior development ##

####################################################################

library(readxl)

prd <- read_excel("pr_data.xlsx") # simulated prior data from the predictions in the survival analysis

(Mielke et al. 2019).

# dataset created using unscaled versions of temp, rh, pH but then scaled for

# models based on scaling of environmental dataset

####################################################################

## Scale Variables temp, rh, and day ##

## In the svtm dataset the scale function is set as scale(center=TRUE, scale = TRUE)

## The value produced in the scaled:scale attribute after scaling svtm variables are use

## in scale= in prd dataset ##

## The value produced in the scaled:center attribute, after scaling svtm variables,

## are used in the center= in prd ##

####################################################################

###Scaling the prd dataset

prd$t<- scale(prd$temp, center = 27.8, scale = 2.33) ##these are the values used in the svtm data when scaled and centered

prd$r<- scale(prd$rh, center = 54.3, scale = 19.2) ##these are the values used in the scaling and centering of data in the svtm data, these have to be rerun

prd$day<-as.numeric(prd$time)

prd$dy<- scale(prd$day, center = 7.17, scale = 7.66) ##these are values in svtm data used to center and scale

#####Subset related to the days that are included in the dataset - pulls only days relevant to what we collected########

prd1<-prd[prd$day %in% c(1.5,4, 14, 17, 20, 21, 29, 30),] ###this sets the days ~equal(no day 1 in this so used 1.5) to env data values

####################################################################

## GLM from dataset created using prediction data to gather priors for mean and variance

## to use in analysis with GLM of data ##

## Interaction not significant, use models surv75 without interaction to collect values

####################################################################

sfmdv <- glm(surv_prob50 ~ dy + t + r + t:r,

family = binomial(link = "logit"),

data = prd1)

summary.glm(sfmdv)

plot(sfmdv)

sf75 <- glm(surv_prob75 ~ dy + t + r + t:r,

family = binomial(link = "logit"),

data = prd1)

summary.glm(sf75)

sf90 <- glm(surv_prob90 ~ day + t + r + t:r,

family = binomial(link = "logit"),

data = prd1)

summary.glm(sf90)

###Trying various probabilities to test effect on t and t:r, checking significance

sfp<- glm(surv_prob50 ~ dy + t + r,

family = binomial(link = "logit"),

data = prd1)

summary.glm(sfp)

####################################################################

#### Models to collect prior mean and sd to use in glm(m) for Area of Effect (Contamination)

####################################################################

surv75 <- glm(surv_prob75 ~ day + r + t,

family = binomial(link = "logit"),

data = prd1)

summary.glm(surv75)

plot(surv75)

surv90 <- glm(surv_prob90 ~ day + r + t,

family = binomial(link = "logit"),

data = prd1)

summary.glm(surv90)

plot(surv90)

##Interaction model to collect priors for a run with a prior on the interaction term

sf75 <- glm(surv_prob75 ~ dy + t + r + t:r,

family = binomial(link = "logit"),

data = prd1)

summary.glm(sf75)

####################################################################

#Collect covariate values (coefficients) for use as priors for data analysis - or as a starting point #

####################################################################

resu_75<- summary.glm(surv75)$coefficients #provides coefficients

write.csv(resu_75, file = "resu_75.csv") #create and save to file

library(broom)

glance(surv75)

resu_surv90<- summary.glm(surv90)$coefficients

write.csv(resu_surv90, file = "resu_surv90.csv")

library(broom)

glance(surv90)

resutr75 <- summary.glm(sf75)$coefficients

write.csv(resutr75, file = "resutr75.csv")

library(broom)

glance(sf75)

####################################################################

## Dataframe for Likelihood and for Jags methods

####################################################################

svtmMod<-data.frame(svtm$fmdv, svtm$fm30, svtm$fm35, svtm$fm40, svtm$Herd, svtm$temp, svtm$rh, svtm$ds, svtm$pth, svtm$dy)

str(svtmMod)

write.csv(svtmMod, file = "svtmMod.csv" )

####################################################################

## The Likelihood of the data from a frequentist GLM and Priors from my lit review for prior

## development

####################################################################

ml<- glm(svtm.fmdv~ svtm.Herd + svtm.rh + svtm.ds, family = binomial, data = svtmMod)

summary(ml)

plot(coefficients(ml)) #

hist(coefficients(ml)) #compare to priorR

m2<- glm(svtm.fmdv~ svtm.Herd + svtm.rh + svtm.ds + svtm.dy + svtm.temp, family = binomial, data = svtmMod)

summary(m2)

plot(coefficients(m2))

hist(coefficients(m2))

####################################################################

## Prior

####################################################################

##### resutr75 - Interaction ##############################

# Estimate Std. Error z value Pr(>|z|) var sd tau

#(Intercept) 2.1025 0.9174 2.292 0.02192 67.3298208 8.20547505 0.01485226

#dy -4.524 1.5525 -2.914 0.00357 192.8205 13.88598214 0.005186171

#t -1.4993 0.7848 -1.91 0.05608 49.2728832 7.019464595 0.020295139

#r 7.5032 2.5265 2.97 0.00298 510.65618 22.59770298 0.001958265

#t:r 0.303 0.6287 0.482 0.6299 31.6210952 5.62326375 0.031624458

#####################################################################

####################################################################

## START HERE TO BEGIN JAGS MODEL WITH DATASET SVTM

####################################################################

library(readxl)

library(rjags)

library(runjags)

svtmC<-read.csv("svtmMod.csv", header = TRUE, sep = ",", dec = ".", na.strings = "NA")

str(svtmC)

svtmM<-na.omit(svtmC)

str(svtmM)

##Index##################### Dummy variables for ds (distance)

epd <- sort(unique(svtmM$svtm.ds))

##Initialize Matrix to hold Dummy Variables##

dsm <- matrix(NA, nrow = nrow(svtmM), ncol = length(epd))

##Loop Method##

for(j in 1: length(epd)){

dsm[ ,j] <- as.integer(svtmM$svtm.ds == epd[j])

}

##Add new Matrix##

dsm2 <- as.data.frame(dsm,colnames(epd))

svtmM2 <- cbind(svtmM, dsm2)

str(svtmM2)

pairs(svtmM2)

write.csv(svtmM2, file = "svtmM2.csv" ) #use this for all models

####################################################################

## JAGS Templates- started with herd as fixed effect

####################################################################

template.jags(svtm.fmdv~svtm.Herd + V2 + V3 + V4 + svtm.rh, data = svtmM2,

file = "jagsvtm.txt", family = "binomial", n.chains = 4)#svtm.ds, using V1, V2, V3, V4 (ds0, 50, 75, 100)

#also tried with quadratic on rh

template.jags(svtm.fmdv~svtm.Herd + V2 + V3 + V4 + svtm.rh + svtm.dy + (svtm.dy^2), data = svtmM2,

file = "jagsvtmdy.txt", family = "binomial", n.chains = 4)#svtm.ds, using V1, V2, V3, V4 (ds0, 50, 75, 100)

####################################################################

### The model fit is best when herd is a random

####################################################################

## Model used to run numerous selection models ## template.jags(svtm.fm40~(1|svtm.Herd) + V1 + V2 + V3 + V4 + svtm.dy + svtm.temp + svtm.rh + svtm.temp:svtm.rh, data = svtmM2, file = "jagshdt.txt", family = "binomial", n.chains = 4) #svtm.ds, using V1, V2, V3, V4 (ds0, 50, 75, 100)

t1<- run.jags(model = "jagshdt.txt") #using as the model with day included

t1

plot(t1)

t2<- run.jags(model = "jagshdtI.txt") #using as the model with day included and with informed prior

t2

plot(t2)

####################################################################

## Checking the results change due to CT threshold change

####################################################################

library(readxl)

svtmM2<-read.csv("svtmM2.csv")

template.jags(svtm.fm30~(1|svtm.Herd) + V1 + V2 + V3 + V4 + svtm.temp, data = svtmM2,

file = "jagsfm30.txt", family = "binomial", n.chains = 4)

jm30<-run.jags(model = "jagsfm30.txt")

jm30

plot(jm30)

template.jags(svtm.fm35~(1|svtm.Herd) + V1 + V2 + V3 + V4 + svtm.temp, data = svtmM2,

file = "jagsfm35.txt", family = "binomial", n.chains = 4)

jm35<-run.jags(model = "jagsfm35.txt")

jm35

plot(jm35)

####################################################################

# CHOSEN - Interaction Model used for interpretation of Data and Prediction

####################################################################

template.jags(svtm.fm40~(1|svtm.Herd) + V1 + V2 + V3 + V4 + svtm.rh + svtm.temp + svtm.temp:svtm.rh, data = svtmM2, file = "jagsRTI.txt", family = "binomial", n.chains = 4)

jm5<- run.jags(model = "jagsRTI.txt")

jm5

plot(jm5$mcmc)

jms<- as.mcmc.list(jm5)

plot(jms)

#Non-informed interaction model

template.jags(svtm.fm40~(1|svtm.Herd) + V1 + V2 + V3 + V4 + svtm.rh + svtm.temp + svtm.temp:svtm.rh, data = svtmM2,

file = "jagsRTN.txt", family = "binomial", n.chains = 4)

jm4<- run.jags(model = "jagsRTN.txt")

jm4

plot(jm4)

out4<-as.jags(jm4)

####################################################################

## The Chosen - both non-informative and informed ##

####################################################################

template.jags(svtm.fm40~(1|svtm.Herd) + V1 + V2 + V3 + V4 + svtm.temp, data = svtmM2,

file = "jagsNIP.txt", family = "binomial", n.chains = 4)

jm1<-run.jags(model = "jagsNIP.txt")

jm1

plot(jm1)

template.jags(svtm.fm40~(1|svtm.Herd) + V1 + V2 + V3 + V4 + svtm.temp, data = svtmM2,

file = "jagsIP.txt", family = "binomial", n.chains = 4)

jm2<-run.jags(model = "jagsIP.txt")

jm2

plot(jm2)

template.jags(svtm.fm40~(1|svtm.Herd) + V1 + V2 + V3 + V4 + svtm.rh, data = svtmM2,

file = "jagsRHN.txt", family = "binomial", n.chains = 4)

jmr<-run.jags(model = "jagsRHN.txt")

jmr

plot(jmr)

template.jags(svtm.fm40~(1|svtm.Herd) + V1 + V2 + V3 + V4 + svtm.rh, data = svtmM2,

file = "jagsRHI.txt", family = "binomial", n.chains = 4)

jmri<- run.jags(model = "jagsRHI.txt")

jmri

plot(jmri)

template.jags(svtm.fm40~(1|svtm.Herd) + V1 + V2 + V3 + V4 + svtm.rh + svtm.temp, data = svtmM2,

file = "jagsENV.txt", family = "binomial", n.chains = 4)

jm6<- run.jags(model = "jagsENV.txt")

plot(jm6)
